# The role of health literacy in explaining the association between educational attainment and the use of out-of-hours primary care services in chronically ill people: a survey study

**DOI:** 10.1186/s12913-018-3197-4

**Published:** 2018-05-31

**Authors:** Tessa Jansen, Jany Rademakers, Geeke Waverijn, Robert Verheij, Richard Osborne, Monique Heijmans

**Affiliations:** 1 NIVEL – Netherlands Institute for Health Services Research, PO Box 1568, 3500 BN, Otterstraat 118-124, 3513 CR Utrecht, The Netherlands; 2 Department of Family Medicine, School for Public Health and Primary Care (CAPHRI), Maastricht University, PO Box 616, 6200 MD Maastricht, the Netherlands; 3 Health Systems Improvement Unit, Centre for Population Health Research, School of Health and Social Development, Deakin University, 221 Burwood Highway, Burwood VIC, Geelong, 3125 Australia; 4 Department of Public Health, The University of Copenhagen, Øster Farimagsgade 5, Postboks 2099, 1014 Copenhagen, Denmark

**Keywords:** Socioeconomic health inequalities, Health literacy, Healthcare use, Out-of-hours, Primary care, Mediation study, Chronic condition

## Abstract

**Background:**

Low socioeconomic status (SES) is persistently associated with poor health and suboptimal use of healthcare services, and more unplanned healthcare use. Suboptimal use of emergency and acute healthcare services may increase health inequalities, due to late diagnosis or lack of continuity of care. Given that health literacy has been associated with healthcare utilisation and with education attainment, we sought to explore whether health literacy is related to the use of out-of-hours (OOH) Primary Care Services (PCSs). Additionally, we aimed to study whether and to what extent health literacy accounts for some of the association between education and OOH PSC use.

**Methods:**

A survey including measures of education attainment, health literacy (assessed by means of the Dutch version of the nine-dimension Health Literacy Questionnaire) and use of PCS was conducted among a sample of adults diagnosed with (any) somatic chronic condition in the Netherlands (response 76.3%, *n* = 1811). We conducted linear and logistic regression analyses to examine associations between education level and PCS use in the past year. We performed mediation analyses to assess whether the association between education and PCS use was (partly) explained by different aspects of health literacy. We adjusted the models for patient characteristics such as age and morbidity.

**Results:**

Higher education attainment was associated with higher scores on the health literacy aspects Appraisal of health information, and Navigating the healthcare system. Additionally, appraisal and navigating the healthcare system partially accounted for educational differences in PCS use. Finally, higher appraisal of health information scores were associated with higher PCS utilisation.

**Conclusion:**

Several aspects of health literacy were demonstrated to relate to PCS use, and partly accounted for educational differences herein. Accordingly, developing health literacy within individuals or communities may help to reduce inappropriate PCS use among people with low education.

## Background

Socioeconomic inequalities in health are persistent and, despite increasing overall wealth, the gap between socioeconomically disadvantaged groups and more affluent groups is widening [1–3]. Individuals who completed higher education are more likely to live in good health than those with lower education [4, 5]. Moreover, low socioeconomic status (SES) relates to lower life expectancy and higher morbidity earlier in life [4, 5]. Once people become ill, chronic conditions are more likely to persist and progress in people with low SES backgrounds compared with more advantaged groups [6, 7]. SES can be operationalized in many different ways, for example by income, neighbourhood, and educational level. In the present study we have chosen for educational level, since it is a powerful indicator and affects other SES indicators such as income and occupation [4, 8–11].

Several potential mechanisms that might cause SES differences related to morbidity and mortality have been studied. For instance, people from low SES backgrounds are more likely to engage in behaviours that are detrimental for health, such as smoking, poor dietary habits, and non-adherence with medication regimens [12, 13]. Low SES is related to greater use of healthcare services, even when higher morbidity is taken into account [14, 15]. Moreover, a social gradient has been demonstrated in the use of acute care through emergency departments and out-of-hours primary care services. Whereas lower SES groups more often turn to emergency and acute care services [16–19], high SES groups tend to use specialist care more often [20]. Unplanned emergency healthcare use is unfavourable in terms of cost control and quality of care than ambulatory care [16, 21]. Furthermore, the use of emergency care jeopardises the continuity of care and patients’ relationships with care providers due to incomplete knowledge of the medical history of the patient [16, 19, 22, 23]. Suboptimal use of these services may therefore reinforce health disparities between socioeconomic groups [16]. In addition, these services may function as an indicator for inadequate provision of care or access to the healthcare system elsewhere [24].

One of the mechanisms underlying SES differences in healthcare use may be found in the concept of health literacy, which captures the difficulties individuals may encounter in finding their way through the healthcare system. By following a social gradient for education, limited health literacy reinforces socioeconomic health inequalities [25]. Health literacy has been hypothesised to be on the pathway between education and health [26, 27]. Nevertheless, although lower health literacy is often observed in people with less education [25], highly educated people may also have poor health literacy skills [26].

Several studies demonstrated that low functional health literacy is related to suboptimal use of healthcare services. Especially with more hospitalizations and greater use of emergency care services (e.g. [28, 29, 30]). Most of these studies have been conducted in the USA, using single-dimension functional health literacy measures. Research to whether the relationship between low health literacy and inopportune healthcare use also exists in other Western healthcare systems is still scarce though. Nevertheless, low health literacy was found to relate to higher healthcare costs in Switzerland and more general practice (GP) use in the Netherlands [31, 32]. Conversely, no association was found in an Australian study between a wide range of health literacy dimensions and readmissions [33].

Health literacy, in general, is defined in many different ways [34, 35]. Most definitions of health literacy focus on functional competencies (reading and numeracy) or on basic skills to obtain and process (written and oral) health information. In order for people to be actively involved in their health and care, more is needed than knowledge and information. Therefore, the importance of taking a more comprehensive perspective on health literacy has been emphasised [34, 36, 37]. Accordingly, an inclusive definition was formulated by the European Health Literacy Consortium: “Health literacy is linked to literacy and entails people’s knowledge, motivation and competences to access, understand, appraise and apply health information in order to make judgements and take decisions in everyday life concerning health care, disease prevention and health promotion to maintain or improve quality of life during the life course.” [25]

**Fig. 1:**
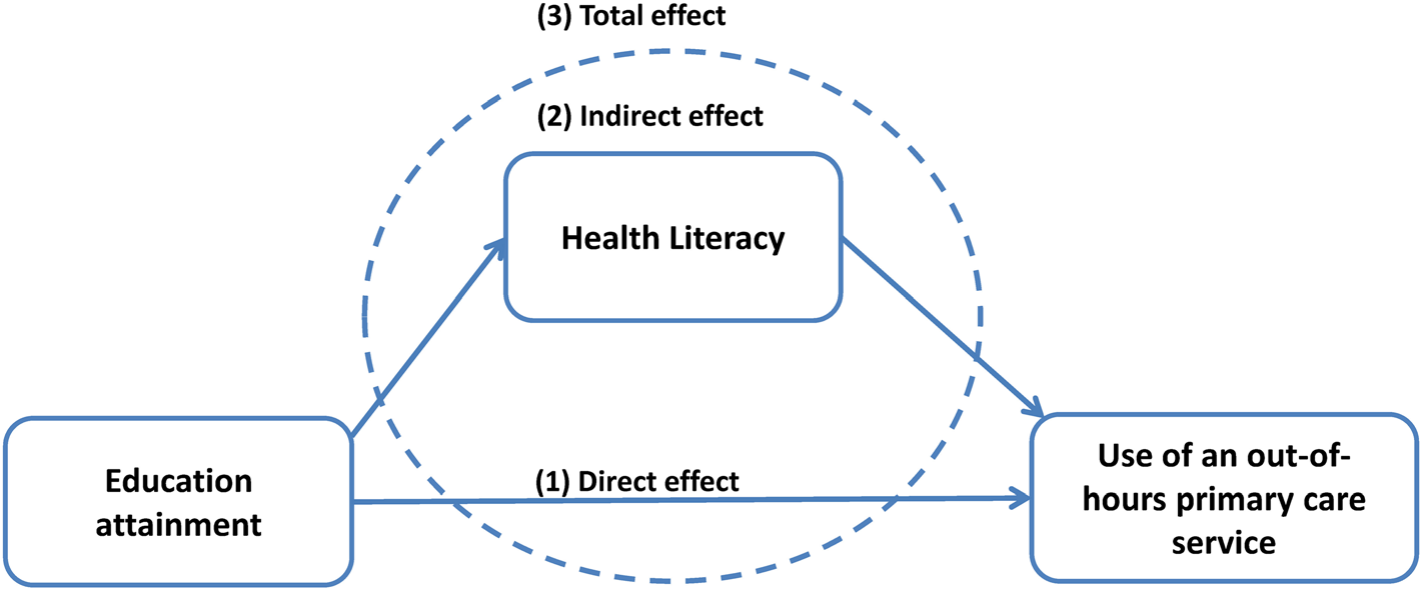
Hypothesised association between education attainment and contact with an out-of-hours primary care service, mediated by health literacy; decomposition of the total effect in direct and indirect effect

People with chronic conditions are increasingly expected to be able to manage their own health [38, 39]. We therefore expect that health literacy in this specific group delineates which patients are prone to educational inequalities in healthcare use. Consequently, the aim of the present paper was to explore whether health literacy relates to the use of out-of-hours (OOH) primary care services in adults with a chronic condition. More specifically, we aim to study whether health literacy explains educational differences in the use of OOH primary care services. We thus assessed the role of health literacy as mediator in the association between education attainment and use of OOH primary care services. The associations we studied are depicted in Fig. 1.

## Methods

### Sample

Data were collected through the *National Panel of People with Chronic Illness or Disability (NPCD)*, covering a nationwide sample of people with somatic chronic diseases and/or physical disabilities [32, 38, 40, 41]. The panel’s purpose is to provide Dutch policymakers information about the impact of chronic illness and disability on daily life and living conditions [40]. Panel members were recruited via a random sample of general practices in the Netherlands, drawn from the Netherlands Register of General Practices [42]. Selection criteria for inclusion were: diagnosed with a somatic chronic disease by a certified medical practitioner, being aware of this diagnosis, aged ≥15 years, not institutionalized, having a life expectancy of at least 6 months according to the GP, being mentally able to participate, being able to read and write the Dutch language. Eligible patients were invited to participate in the panel, for a maximum of four years, and receive a self-report questionnaire biannually. The NPCD is registered with the Dutch Data Protection Authority, and all data were collected and handled according Dutch privacy legislation.

For the present study, data were derived from the 2014 spring survey. A questionnaire was sent in April to 2375 NPDC panel-members diagnosed with at least one somatic chronic disease, and completed by 1811 people (response rate 76.3%). Non-response analysis on relevant background characteristics (sex, age group, education level, household status, and number and type of chronic diseases) indicated that non-responders, compared to responders, were slightly younger, and were slightly more often diagnosed with one chronic disease in contrast with more than one. Moreover, they were somewhat more often diagnosed with respiratory – and neurological diseases, and less often diagnosed with cardiovascular disease and diabetes.

### Measures

#### Use of out-of-hours primary care service

As outcome variable we used one item that assessed whether a person used an out-of-hours primary care service (PCS) in the past year. The dichotomous variable was coded 1 for having used, and 0 for not having used a PCS.

#### Education attainment

Education attainment was assessed as the highest level of education the respondent attained, grouped in three categories consistent with the International Standard Classification of Education [43]: low (no education, primary school only, lower vocational education), intermediate (intermediate or advanced general education, intermediate vocational education), and high (higher professional education, university). In the analyses, low education served as reference category.

#### Health literacy

To assess health literacy, we used the Health Literacy Questionnaire (HLQ) developed by Osborne et al. [44] (Dutch version of Heijmans et al.). The HLQ measures the full breadth of the health literacy concept. Consequently, across nine scales (each with 4 to 6 items) the HLQ captures a broad array of skills, cognitions and beliefs of individuals and their experiences of engaging with services. Table 1 briefly describes the nine scales of the HLQ; for a more comprehensive explanation of the meaning of each of the scales, see Osborne et al. [44]. The scales were found to have strong construct validity and the be highly reliable [44–47].

**Table 1:** Nine scales of the Health Literacy Questionnaire [44]

Scales	
1. Feeling understood and supported by health care providers *(1 = strongly disagree; 4 = strongly agree)* 2. Having sufficient information to manage my health *(1 = strongly disagree; 4 = strongly agree)* 3. Actively managing my health *(1 = strongly disagree; 4 = strongly agree)* 4. Social support for health *(1 = strongly disagree; 4 = strongly agree)* 5. Appraisal of health information *(1 = strongly disagree; 4 = strongly agree)* 6. Ability to actively engage with health care providers *(1 = cannot do; 5 = very easy)* 7. Navigating the health care system *(1 = cannot do; 5 = very easy)* 8. Ability to find good health information *(1 = cannot do; 5 = very easy)* 9. Understand health information enough to know what to do *(1 = cannot do; 5 = very easy)*	

The scales grade on an ordinal scale from 1 = *strongly disagree* to 4 = *strongly agree* for scale one to five (Table 3), and from 1 = *cannot do* to 5 = *very easy* for scale six to nine. Scale scores were computed by summing the values of each item and dividing the score by the number of items. Higher scores reflect better health literacy. Missing values on single items ranged from 3.8 to 6.4%. Four to five item scales with a maximum of two missing values, and the six item scale with a maximum of three missing values were imputed using the expectation maximization (EM) algorithm in SPSS version 21 [48, 49]. We excluded respondents who had more missing items than could be imputed from further analyses.

#### Background characteristics

Background characteristics of the respondents include age (continuous variable in main analyses, and in age groups for descriptive analysis: 15–39 years, 40–64 years, 65–74 years, and 75 and older), sex, household status (living with or without a partner), type and number of medically diagnosed chronic diseases (one, two, three or more), and illness duration in years (time since the first diagnosis of a chronic disease). In case a patient had been diagnosed with more than one chronic disease, only the first diagnosed type of disease was taken into account for descriptive purpose and calculation of illness duration. The type of disease was based on ICPC codes (International Classification of Primary Care, version 1 [50, 51]), derived from the routine electronic health records kept by the patient’s General Practitioner. We grouped chronic diseases into eight disease categories (Table 1).

### Statistical analyses

We used descriptive statistics to describe the sample. We performed bivariate linear and logistic regression models to examine whether there was an association between the outcome variable having used a PCS in the past year and education level, and between the outcome and the nine distinct scales of health literacy. Subsequently, we performed mediation analyses for the HLQ scales that we found to be significantly associated with the outcome variable. Mediation occurs when the independent variable affects the outcome variable through another variable, the mediator [52]. Since classical methods, such as Baron & Kenny’s method, use linear models to assess mediation (e.g. [53]), these are difficult to interpret for logit models. Therefore, we used the *KHB* method for Stata [54], which was developed for application in logit and probit regression models. The KHB method allows comparisons between the estimated coefficients, even though variables that are included in the models are measured on different scales (e.g. continuous, ordinal) [55, 56]. We used the KHB method to separately estimate the total effect of the independent variable education level on the outcome OOH primary care use. The total effect was divided in a direct effect of the independent variable socioeconomic status and indirect effect of the mediator health literacy (Fig. 1).

Figure 1 depicts the assumptions that have to be met to establish mediation: (1) The association between the independent variable *education level* and the outcome variable *use of an OOH PCS* should be statistically significant (total effect); (2) Both the associations between the independent variable *education level* and the mediator *health literacy*, and the association between the mediator *health literacy* and the outcome *use of a PCS* should be statistically significant (together these associations constitute the indirect effect of education on the use of an OOH PCS); (3) The association between the independent variable, mediated through the mediator, and the outcome variable should be statistically significant (total effect). For mediation to occur, the mediator should (at least partly) account for the total effect of the independent variable on the outcome (indirect effect). The direct effect of the independent variable on the outcome should diminish or disappear after entering the mediator. Besides direct, indirect and total effect, the KHB mediation analysis results in a confounding ratio, and a confounding percentage. The former refers to the contribution of the model with the mediator compared to the model without the mediator. That is, the size of the total effect (the coefficients from the effect of education on OOH primary care use, mediated through health literacy) divided by the direct effect (the coefficients from the effect of education on OOH primary care use). The latter refers to the percentage of the model effect that is attributable to the mediator [56, 57].

Initially, we applied models unadjusted for sociodemographic characteristics to assess whether mediation occurred. Successively, we adjusted the mediation models for these characteristics. We conducted statistical analyses using Stata version 14.0 [58]. We considered results to be statistically significant if the *p*-value was < 0.05.

## Results

### Characteristics of the sample

Table 2 depicts the sample characteristics. Mean age of the respondents was almost 63 years (SD 14.0, range 15–92 years), and 53% was female. Most respondents attained intermediate education level, and the majority lived together with a partner. Illness duration ranged from half a year up to 66 years (mean almost 13 years). Respiratory disease was the most frequently diagnosed first chronic condition (in case a patient suffered from more than one chronic condition). 53% suffered from more than two chronic diseases. Almost 20% of the respondents used a PCS in the past year.

**Table 2:** Descriptive statistics of the sample of people with chronic disease

		% or mean (SD)
		(n = 1811)
Age in years		62.9 (14.0)
Sex	Male	46.7
	Female	53.3
Age group in years	15–39	7.3
	40–64	40.4
	65–74	32.8
	75 and older	19.5
Education	Low	31.0
	Intermediate	42.2
	High	24.4
	Unknown	2.4
Household status	Living alone	27.1
	Living together	72.0
	Unknown	0.9
First diagnosed chronic condition	Cardiovascular disease	15.6
	Respiratory disease	37.3
	Musculoskeletal disease	10.4
	Cancer	4.8
	Diabetes	11.0
	Neurological disease	5.0
	Digestive disease	4.1
	Unspecified other disease	11.9
Number of chronic conditions	One	46.8
	Two	30.7
	Three or more	22.5
Illness duration in years (*n* = 1783)		12.8 (9.9)
used an out-of-hours primary care service in the past year	No	75.8
	Yes	19.7
	Unknown	4.5

### Education and OOH primary care use

Table 3 illustrates mean scale scores of the nine HLQ scales. In addition it shows odds ratios between education and having contacted a PCS in the past year, and odds ratios between HLQ scales and having contacted a PCS. The table shows associations, both unadjusted and adjusted, for sociodemographic characteristics. The association between education and OOH primary care use (Fig. 1, path 1) was significant, and remained significant after adjusting for sociodemographic characteristics (Table 3). More low educated people used a PCS (26.3%) than the intermediate (17.8%) and high educated (18.3%).

**Table 3:** Mean HLQ scale scores and odds ratios (OR) for bivariate associations between education and having used an out-of-hours primary care service in the past year, and HLQ scales and having used a primary care out-of-hours service in the past year

		Used an out-of-hours primary care service^a^ (unadjusted)	Used an out-of-hours primary care service^a^ (adjusted)^b^
	Mean scale score (SD)	OR (95 CI)^c^	OR (95 CI)^c^
Education			
Low (ref)	–	1.00	1.00
Intermediate	–	0.61 (0.46; 0.80)***	0.65 (0.49; 0.85)**
High	–	0.63 (0.46; 0.86)**	0.70 (.50; 0.96)*
Number of chronic conditions			
One	–	1.00	–
Two	–	1.09 (0.83; 1.44)	–
Three or more	–	1.37 (1.02; 1.83)*	–
Illness duration in years	–	1.01 (1.00; 1.02)*	–
HLQ scale^d^			
Feeling understood and supported by healthcare providers (range 1–4) (*n* = 1745)	2.97 (.51)	1.14 (0.90; 1.45)	1.12 (0.88; 1.43)
Having sufficient information to manage my health (range 1–4) (*n* = 1741)	2.92 (.42)	0.83 (0.63; 1.10)	0.81 (0.61; 1.08)
Actively managing my health (range 1–4) (*n* = 1723)	2.85 (.43)	1.29 (0.98; 1.70)	1.31 (0.99; 1.74)
Social support for health (range 1–4) (*n* = 1739)	2.91 (.49)	0.87 (0.68; 1.11)	0.88 (0.69; 1.13)
Appraisal of health information (range 1–4) (*n* = 1724)	2.64 (.48)	1.43 (1.11; 1.84)**	1.52 (1.18; 1.97)**
Ability to actively engage with healthcare providers (range 1–5) (*n* = 1709)	3.97 (.62)	0.76 (0.63; 0.92)**	0.79 (0.65; 0.96)*
Navigating the healthcare system (range 1–5) (*n* = 1720)	3.87 (.62)	0.75 (0.63; 0.91)**	0.79 (0.65; 0.95)*
Ability to find good health information (range 1–5) (n = 1709)	3.90 (.62)	0.71 (0.59; 0.85)***	0.76 (0.62; 0.92)**
Understanding health information well enough to know what to do (range 1–5) (*n* = 1711)	4.03 (.57)	0.73 (0.60; 0.89)**	0.76 (0.62; 0.94)*

^a^ Reference is not having used a primary in out-of-hours service in the past year

^b^ Adjusted for age (continuous), sex (ref male), household status (ref living alone), number of chronic diseases (ref one chronic disease), and illness duration

^c^ *P*-value * < 0.05, ** < 0.01, *** < 0.001, significant results printed in bold

^d^ Higher scores on the HLQ scales reflect better health literacy

### Education and health literacy

The health literacy scale scores tended to cluster around the upper half of the scales, with highest scores on *Feeling understood* and *Understanding health information*. Additionally, education level was significantly associated with six out of nine health literacy scales (Fig. 1, path 2), where higher education was associated with higher health literacy scores (Table 4). After adjusting for sociodemographic characteristics, the association remained significant (Table 5). We did not find significant associations between education level and the HLQ scales *Feeling understood*, *Actively managing health*, and *Social support for health*.

**Table 4:** Unadjusted coefficients (B) for bivariate associations between education and HLQ scales, linear regression

	Feeling understood and supported by healthcare providers (*n* = 1704)	Having sufficient information to manage my health (*n* = 1700)	Actively managing my health (n = 1682)	Social support for health (*n* = 1698)	Appraisal of health information (*n* = 1683)	Ability to actively engage with healthcare providers (*n* = 1669)	Navigating the healthcare system (*n* = 1680)	Ability to find good health information (*n* = 1669)	Understanding health information well enough to know what to do (*n* = 1671)
	B (95% CI)^a^	B (95% CI)^a^	B (95% CI)^a^	B (95% CI)^a^	B (95% CI)^a^	B (95% CI)^a^	B (95% CI)^a^	B (95% CI)^a^	B (95% CI)^a^
Education									
intermediate	0.03 (− 0.03; 0.09)	0.06 (0.02; 0.11)**	0.02 (− 0.02; 0.07)	0.00 (− 0.06; 0.05)	0.05 (− 0.01; 0.10)	0.17 (0.10; 0.24)***	0.20 (0.13; 0.27)***	0.26 (0.20; 0.33)***	0.25 (0.19; 0.31)***
High	0.04 (−0.03; 0.10)	0.09 (0.04; 0.14)**	0.01 (−0.04; 0.07)	0.01 (−0.05; 0.07)	0.11 (0.05; 0.17)***	0.32 (0.24; 0.40)***	0.29 (0.21; 0.37)***	0.40 (0.33; 0.48)***	0.44 (0.37; 0.51)***

^a^ *P*-value * < 0.05, ** < 0.01, *** < 0.001, significant results printed in bold

^b^ Reference is low education

**Table 5:** Adjusted coefficients (B) for bivariate associations between education and HLQ scales^a^, linear regression

	Feeling understood and supported by healthcare providers (*n* = 1676)	Having sufficient information to manage my health (*n* = 1672)	Actively managing my health (*n* = 1682)	Social support for health (*n* = 1670)	Appraisal of health information (*n* = 1656)	Ability to actively engage with healthcare providers (*n* = 1642)	Navigating the healthcare system (*n* = 1651)	Ability to find good health information (n = 1642)	Understanding health information well enough to know what to do (n = 1642)
	B (95% CI)^b^	B (95% CI)^b^	B (95% CI)^b^	B (95% CI)^b^	B (95% CI)^b^	B (95% CI)^b^	B (95% CI)^b^	B (95% CI)^b^	B (95% CI)^b^
Education^c^									
intermediate	0.04 (−0.02; 0.09)	0.05 (0.01; 0.10)*	0.01 (−0.04; 0.06)	−0.01 (− 0.06; 0.05)	0.02 (− 0.03; 0.08)	0.14 (0.07; 0.21)***	0.16 (0.09; 0.23)***	0.20 (0.13; 0.27)***	0.20 (0.14; 0.26)***
High	0.04 (−0.02; 0.11)	0.09 (0.03; 0.14)**	0.00 (−0.06; 0.06)	0.01 (−0.06; 0.05)	0.09 (0.03; 0.15)**	0.29 (0.21; 0.37)***	0.25 (0.17; 0.33)***	0.34 (0.26; 0.42)***	0.39 (0.32; 0.46)***

^a^ All models are adjusted for age (continuous), sex (ref male), household status (ref living alone), number of chronic diseases (ref one chronic disease), and illness duration

^b^ *P*-value * < 0.05, ** < 0.01, *** < 0.001, significant results printed in bold

^c^ Reference is low education

### Health literacy and OOH primary care use

Individuals who used a PCS scored slightly lower on six HLQ scales than those who did not use a PCS. For instance, the mean score for individuals who used a PCS for *Navigating the health care system* was 3.78, whereas the mean score was 3.89 for those who did not. The opposite was observed for the scales *Feeling understood*, *Actively managing*, and *Appraisal* (not shown)*.*

From the HLQ scales one to five (Table 1), only *Appraisal of health information* was significantly associated with OOH primary care use (Table 3; Fig. 1, path 2). However, this association was inverse: a higher score on the *appraisal* scale was associated with having used a PCS more often. Scales six to nine (Table 1) were significantly associated with the outcome. These included health information seeking and understanding, finding the way to health services and the ability to obtain preferred healthcare services. The direction of the association was negative: higher scores were associated with having used a PCS less often. The associations remained significant after adjusting for sociodemographic characteristics (Table 3). The support scales, i.e. *Feeling understood* and *Social support for health*, were not significantly associated with use of PCSs. Similarly, confidence about having sufficient information to make decisions, and the recognition of own responsibility for their health were not significantly related.

### Education and OOH primary care use mediated through health literacy

Mediation analyses were justified for five scales (Fig. 1, path 3). Table 6 shows that all five health literacy scales significantly mediated the association between education and use of a PCS, unadjusted for background characteristics. We found the largest mediating effect for *Ability to find good health information*. The total effect of education on OOH primary care use, mediated through *Ability to find good health information,* was 1.3 times larger than the direct effect, with almost 23% of the total effect attributable to *Ability*. Three other HLQ scales mediated about 12 to 20% of the total effect, and enlarged the direct effects with factors ranging from 1.14 to 1.24.

**Table 6:** Unadjusted odds ratios (OR) for direct, indirect and total effects for the association between education and out-of-hours primary care use, mediated through health literacy, using the KHB method with logistic regression

	Appraisal of health information (*n* = 1638)	Ability to actively engage with healthcare providers (*n* = 1629)	Navigating the healthcare system (*n* = 1639)	Ability to find good health information (n = 1629)	Understanding health information well enough to know what to do (*n* = 1636)
	OR (95% CI)^a^	OR (95% CI)^a^	OR (95% CI)^a^	OR (95% CI)^a^	OR (95% CI)^a^
Total effect (education and HLQ)	0.78 (0.66; 0.92)**	0.78 (0.66; 0.92)**	0.76 (0.65; 0.89)**	0.77 (0.66; 0.91)**	0.77 (0.65; 0.90)**
Direct effect (education)	0.76 (0.65; 0.90)**	0.81 (0.68; 0.95)*	0.79 (0.67; 0.93)**	0.82 (0.69; 0.97)*	0.81 (0.68; 0.96)*
Indirect effect (HLQ)	1.02 (1.00; 1.04)*	0.97 (0.94; 1.00)*	0.97 (0.94; 1.00)*	0.94 (0.90; 0.98)**	0.95 (0.90; 1.00)*
Confounding ratio (total effect/direct effect)	0.92	1.16	1.14	1.30	1.24
Confounding percentage (% of model affect attributable to mediator)	−8.90%	13.78%	12.27%	22.88%	19.65%
Pseudo R square ^b^	0.01	0.01	0.01	0.01	0.01

^a^ *P*-value * < 0.05, ** < 0.01, *** < 0.001, significant results printed in bold

^b^ According to McFadden method [51]

Although *Appraisal of health information* significantly mediated the association between education and use of OOH primary care as well, the mediated effect counteracted with the effect of education. The effect of *Appraisal* as mediator could be explained by the different directions of the associations. i.e., higher education was associated with less frequently using a PCS (−). Moreover, higher education was related to higher scores on the appraisal scale (+). However, higher appraisal was associated with more OOH primary care use (+). Consequently, appraisal as mediator acted as suppressor for the direct effect from education on OOH primary care use (Fig. 1, path 1). Inasmuch as the indirect effect (Fig. 1, path 2) induced an underestimation of the total effect (Fig. 1, path 3), as expressed by the confounding ratio of 0.92.

Table 7 depicts the mediation models, adjusted for sociodemographic characteristics, for example age, sex, and disease. After adjusting, two of the HLQ scales remained significant mediators, i.e. A*ppraisal of health information* and *Navigating the health care system*. The inverse effect of *appraisal* remained. With regard to *Navigating the health care system*, the total effect of education and navigating the healthcare system on OOH primary care use was 1.13 larger than the direct effect of education alone. Additionally, 11.8% of the effect could be ascribed to navigating the health care system.

**Table 7:** Adjusted odds ratios (OR) for direct, indirect and total effects for the association between education and out-of-hours primary care use, mediated through health literacy, using the KHB method with logistic regression^a^

	Appraisal of health information (*n* = 1612)	Ability to actively engage with healthcare providers (*n* = 1603)	Navigating the healthcare system (n = 1612)	Ability to find good health information (n = 1603)	Understanding health information well enough to know what to do (*n* = 1609)
	OR (95% CI)^b^	OR (95% CI)^b^	OR (95% CI)^b^	OR (95% CI)^b^	OR (95% CI)^b^
Total effect (education and HLQ)	0.82 (0.69; 0.98)*	0.82 (0.69; 0.97)*	0.80 (0.68; 0.95)*	0.82 (0.69; 0.97)*	0.81 (0.69; 0.96)*
Direct effect (education)	0.81 (0.68; 0.96)*	0.85 (0.71; 1.01)	0.83 (0.70; 0.98)*	0.85 (0.72; 1.01)	0.85 (0.71; 1.01)
Indirect effect (HLQ)	1.02 (1.00; 1.04)*	0.97 (0.94; 1.00)	0.97 (0.95; 1.00)*	0.96 (0.92; 0.99)*	0.96 (0.92; 1.00)
Confounding ratio (total effect/direct effect)	0.91	1.18	1.13	1.27	1.25
Confounding percentage (% of model affect attributable to mediator)	−9.79%	14.92%	11.83%	21.07%	19.91%
Pseudo R square ^c^	0.02	0.02	0.02	0.02	0.02

^a^ All models are adjusted for age (continuous), sex (ref male), household status (ref living alone), number of chronic diseases (ref one chronic disease), and illness duration

^b^ *P*-value * < .05, ** < .01, *** < .001, significant results printed in bold

^c^ According to McFadden method [51]

## Discussion

### Main findings

The aim of the present study is to explore whether health literacy is related to the use of out-of-hours primary care services (PCSs) by people with chronic conditions. We expected to find that individuals with higher health literacy less often used PCSs. In our sample, PCSs were attended by more patients than in the general population: 20% compared to 15% of the Dutch population in 2013 (NIVEL Primary Care Database [59]). Due to our sample of people with a chronic disease and/or disability, more healthcare use was expected.

We assessed health literacy using the Health Literacy Questionnaire (HLQ) [44]. We demonstrated significant associations between *Appraisal of health information*¸ *Ability to actively engage with healthcare providers*, *Navigating the healthcare system*, *Ability to find good health information*, and *Understanding health information well enough what to do*. For the latter four aspects, higher levels of health literacy was related to less use of PCSs. We however observed that higher *Appraisal of health information* was associated with more use of PCSs.

Our results compare with previous studies that linked lower health literacy to visiting a GP more often [32], and to more use of the Emergency Department [30, 60]. Whereas these studies merely indicated an association for functional health literacy, our study provides evidence for a broader set of health literacy skills and capabilities.

The second objective was to determine whether health literacy could explain educational differences in the use of PCSs. Higher education level was significantly related to higher health literacy. Correspondingly, Beauchamp et al. [61] demonstrated significant associations between education level and three aspects that we also found to be associated with education. In general, health literacy is frequently demonstrated to be correlated with education [25, 29]. Nevertheless, low levels of health literacy were also found among people with higher education [26, 62]. Subsequently, we demonstrated that five aspects of health literacy accounted for educational differences in PCS use. The *Ability to find good health information* and *Understanding health information well enough what to do* accounted the most for educational differences. After adjusting for sociodemographic characteristics, the effects of three of the unadjusted models were substantially reduced though. Similar to our study, age was found to be highly related to both use of PCSs [63], health literacy [38, 61], and education attainment [8].

Whereas *Navigating the health care system* mediated about 12% of the education effect after adjustment, *Appraisal of health information* induced an compensatory effect of the association between education and OOH primary care use, due to opposing mediation [57]. Although higher educated individuals obtained higher appraisal scores, higher appraisal was related to more use of PCSs. Better appraisal of health information was also found to be related to more involvement in medical decision-making [64]. Perhaps, in our sample, appraisal reflected being more critical in evaluating health information because of being more experienced in – and knowledgeable about healthcare use. Therefore, the effect of lower education on OOH primary care use could have been compensated for by the ability to appraise health information. This finding could lead to inferences about interventions to reduce inequalities in healthcare use. If people could be supported to make good decisions by appraising well, they may be able to avoid PCS use, despite being low educated.

Our study indicated that PCS use is related to information seeking and evaluating, and to finding the way to the designated healthcare providers and engage with them. Conceivably, being able to find and understand health information may prevent someone attending acute health services out of a sense of insecurity about their health condition. In addition, someone might not turn too quickly to the easily accessible PCS, when he or she is able to find their way through the healthcare system and engage with healthcare providers. In contrast, the other health literacy aspects seemingly reflect more day-to-day skills and resources that do not clearly differ between subgroups, nor relate specifically to PCS use. The former was analogous to the findings of Beauchamp et al. [61].

### Strengths and weaknesses

A strength of this study is the use of the Health Literacy Questionnaire (HLQ), since this robust measure represents the full breadth of health literacy, and goes beyond merely functional skills [44]. To our knowledge, the present study is the first to relate the HLQ to healthcare use in primary care [33].

The present study has some limitations. The cross-sectional design of the study does not allow for conclusions about causality. Education has been proven to be a powerful determinant of socioeconomic differences in health behaviours, outcomes and mortality [4, 9, 10], and affects other SES determinants such as income and occupation [65]. Moreover, income as operationalisation of SES probably does not do justice to the association under study, since primary care is covered by insurance for everyone in the Netherlands [66, 67].Nevertheless, education attainment may have underestimated the actual cognitive ability of the respondents in the sample. If respondents in our sample already suffered from a chronic condition in their childhood, they may not have been able to attend education at a level that justified their cognitive ability. This may have resulted in a selection effect [8]. Additionally, education is related to birth cohorts, as younger age groups have easier access to education.. In future studies, a more comprehensive measurement of socioeconomic status, such as a composite individual, household, and neighbourhood level indicator [8] may overcome this effect.

In the present study health literacy was assessed by means of a paper-based questionnaire, which by design, excluded illiterate individuals. We therefore did not include the most vulnerable individuals. Notwithstanding this, the response rate was high, and many people with low health literacy and low education were able to take part. The questionnaire was cognitively tested, also among highly disadvantaged people with low education [68]. Additionally, respondents were invited to access support to complete the questionnaire, however, only 5,2% of respondents used this service. Moreover, our sample resembled the Dutch population regarding health literacy levels that were assessed by means of oral interviews [69]. Another limitation of the use of a questionnaire is the measurement of self-reported use of PCSs, which may have induced recall bias. Future studies may consider interview-based assessment to minimise these limitations.

By looking at one scale at the time, we did not account for different combinations of health literacy people may have had. For example,they may have poor understanding, but have strong social support. Future research should explore how different combinations of health literacy aspects predict future healthcare use.

In our sample, health literacy scores were skewed to the right (high score), as was observed by Beauchamp as well [61]. Consequently, it was difficult to discern the scores between high and low educated subgroups within the sample. Finally, we did not include health status as confounder for healthcare use. Thus, we potentially overestimated the association between education level and healthcare use. Nevertheless, lower education is related to low self-reported health status [10, 70]. Similarly, low health literacy has been associated with low self-reported health status [26]. In addition, health status is partly taken into account by controlling for morbidity.

### Health policy implications

Although our evidence for health literacy as mediator for educational differences in OOH primary care use is limited, we do want to make a case for the need to better understand the role of health literacy in explaining educational differences in healthcare use. Whether more highly educated groups have access to information and the resources needed to take action to prevent disease or achieve better health outcomes, groups with low education often lack these resources [2, 3]. These resources could be developed within individuals, organisations, the community, and the healthcare system by strengthening health literacy. Moreover, better health literacy is easier to attain than a higher education level, and as such it is a promising concept to reduce health inequalities [25]. Since poor health literacy is not limited to the lower educated [26], strengthening health literacy in the general population would not only benefit the lower educated, the higher educated are likely to take advantage as well. Accordingly, policy interventions to increase health literacy may well turn out to be cost-effective by guiding people to the appropriate healthcare provider, and thus fostering adequate healthcare resource allocation. Our findings suggest that the efforts should primarily focus on health literacy interventions facilitating patients to play a more active role by helping them to better retrieve and understand health information, engage with healthcare providers, and finding their way in the healthcare system, rather than merely improving their functional health literacy.

## Conclusion

The present study supports that health literacy is a promising determinant of the use of out-of-hours primary care services (PCSs). Consequently, our study demonstrates that several aspects of health literacy could be developed within people with chronic conditions to avoid inappropriate use of PCSs. Moreover, we found support that two aspects of health literacy account for educational differences in the use of PCSs. Educational differences in PCS use therefore could partly be overcome by strengthening health literacy. This may be through provision of resources to guide decisions regarding seeking appropriate healthcare providers. For the other aspects of health literacy, mediation of the associations between education and use of PCSs declined after including for example age and morbidity.

To be able to devise interventions that eventually reduce health inequalities, we need to better understand the (causal) pathways between education and broader indicators of social position, health literacy, healthcare use and health outcomes. In addition, we need to understand the effect of health literacy on healthcare use of other disciplines, such as specialist care. Furthermore, we need to have a better understanding of the cost-effectiveness of health literacy interventions.

## Abbreviations

EM: Expectation Maximisation
GP: General Practice
HLQ: Health Literacy Questionnaire
ICPC: International Classification of Primary Care
NPCD: National Panel of People with Chronic Illness or Disability
OOH: out-of-hours
PCS: Primary Care Service
SES: Socioeconomic Status
